# Influence of Employment Conditions and Length of Residence on Adherence to Dietary Recommendations in Immigrant Workers in Spain

**DOI:** 10.3390/ijerph15112488

**Published:** 2018-11-08

**Authors:** Ikram Benazizi, Elena Ronda-Pérez, Rocío Ortíz-Moncada, José Miguel Martínez-Martínez

**Affiliations:** 1 Preventive Medicine and Public Health area, Faculty of Health Science, University of Alicante, 03690 Alicante, Spain; ikram.benazizi@ua.es (I.B.); rocio.ortiz@ua.es (R.O.-M.); jmiguelmartinezmartinez@gmail.com (J.M.M.-M.); 2 Immigration and Health Program, CIBERESP, 28029 Madrid, Spain; 3 Research Group on Food and Nutrition (ALINUT), University of Alicante, 03690 Alicante, Spain; 4 Research and Analysis Service IT/EP, MC Mutual, 08037 Barcelona, Spain; 5 Department of Statistics, Technical University of Catalonia, 08028 Barcelona, Spain

**Keywords:** food consumption, diet, Spain, acculturation, occupational health, migration

## Abstract

The objective of this article is to analyze the influence of employment conditions on adherence to dietary recommendations among those born in Spain and immigrants by their time of residence. Data were used from the Platform of Longitudinal Studies of Immigrant Families (PELFI) cohort (*n* = 215) to compare Spaniards and immigrants with <14 and >14 years of residence. The questionnaire on frequency of food consumption (15 items) was used to measure adherence to dietary recommendations. Logistic regression models were used, adjusting for sociodemographic characteristics and employment conditions. Adherence to dietary recommendations was greater among Spaniards, followed by immigrants with >14 years of residence and <14 years of residence. The greatest adherence among Spaniards was for eggs (immigrants ≥ 14 years: 1/ORa = 2.89, <14 years: 1/ORa = 3.92), fish (immigrants ≥ 14 immigrants: 1/ORa = 2.33, <14 years: 1/ORa = 4.72), vegetables (immigrants ≥ 14 years: 1/ORa = 3.26, <14 years: 1/ORa = 4.87), dairy products (immigrants ≥ 14 years: 1/ORa = 14.34, <14 years: 1/ORa = 26.78), and sugary drinks (immigrants ≥14 years: 1/ORa = 2.12, <14 years: 1/ORa = 3.48), and the lowest adherence was for the consumption of sausages and cold cuts (immigrants ≥ 14 years: Ora = 7.62, <14 years: ORa = 24.65). Adjusting for sociodemographic and employment conditions variables did not result in variation in the observed differences between Spaniards, immigrants with <14 years of residence, and immigrants with >14 years of residence.

## 1. Introduction

Employment conditions are important determinants of nutritional behavior in workers. For example, shift work during irregular and nighttime hours has been associated with changes in the circadian distribution of food ingestion [1] and with the presence of chronic health issues such as obesity, cardiovascular diseases, and type II-diabetes [2]. Prior studies have shown that this type of workday is related to unhealthy eating [3,4], characterized by unhealthy dietary behavior such as irregular meals, scarce warm meals during the day [5], consumption of low nutrient foods, and less availability of and access to healthy foods [6]. Long working hours are also another employment condition that has been associated with unhealthy eating habits such as skipping meals and consumption of fast food [7], especially due to a lack of time to prepare meals [8]. Income can also modify the quality of one’s diet and state of nutrition, increasing the risk of a deficient diet [9], with unhealthy eating behavior such as consumption of sugary drinks [10].

Immigrant workers are a group characterized by working conditions that include long work days, low wages, and irregular hours [11]. However, studies of the nutrition habits and working conditions of immigrants are scarce. An analysis of the MICASA cohort (investigation of occupational and environmental health risks of Latino agricultural workers in California) showed low adherence to dietary recommendations for the consumption of fruit, vegetables, and fats [12]. Sliwa et al., using a national survey of Hispanic women in the United States, also showed that long work days reduced the time dedicated to food preparation. This fact made consumption of homemade meals more difficult, with women increasingly resorting to prepared foods (potentially of lower nutritional quality) and more frequent dining at fast food restaurants [8].

In addition to the research related to immigrant nutrition, there is also the effect of “dietary acculturation” [13], a process in which immigrants adopt the dietary habits and choices of the host country, giving way to dietary changes that can be healthy or unhealthy. A systematic review of studies carried out in the United States concluded that immigrants with lower levels of acculturation (measured through different indicators) consumed more fruit, rice, and beans, and less sugar and sugary drinks [14].

A study with Latinos residing in the United States shows that greater levels of acculturation negatively affected adherence to dietary recommendations [12], and acculturation was associated with lower consumption of beans and peas, and greater consumption of prepared foods and sweets, salty snacks and high-fat foods [15].

Changes in dietary habits of Latin American immigrants in Europe, especially in Spain, include greater consumption of refined foods and complex carbohydrates, which are associated with the consumption of easy-access, processed foods [16]. Spain is the fourth largest country in Europe in terms of the proportion of immigrants [17], at 11% of the population. Among immigrants, South Americans are the most represented group, especially those from Colombia and Ecuador [18]. A study of Ecuadorians and Colombians in Spain showed that they maintained a balanced consumption of immediate principles, and an adequate adherence to the recommendations of the Ministry of Agriculture, Fishery and Foods, in terms of eggs, dairy foods and legumes. Despite this, there was insufficient consumption of a few basic food groups, including grains, potatoes, vegetables, and fruit. Spaniards consumed more meat, cereals, vegetables, legumes and soft drinks, and they consumed less fish and fruits. The study concluded that time of residence could be a factor in the pattern of adaptation [19]. Another study of Ecuadorian immigrants in Spain showed that they had better dietary habits than those living in Ecuador [20]. In general, the results of these studies are consistent in terms of the low adherence to dietary recommendations for fruits, vegetables, and fish [21]. However, they are not consistent in terms of the effect of the time of residence in Spain, given that there are both healthy and unhealthy changes in the dietary habits and eating patterns among Latin American immigrants [22]. This is despite the Mediterranean diet in Spain being characterized by healthy eating habits [23]. None of these studies evaluated the relationship between employment conditions and dietary habits in these immigrants.

Therefore, the objective of this study is to analyze the influence of employment conditions on adherence to dietary recommendations among Latin American immigrants by their time of residence in Spain, compared to native Spaniards.

## 2. Materials and Methods

### 2.1. Design

An epidemiological study was undertaken using personal interviews with immigrant and Spaniard families in Spain in Alicante and Barcelona from the Platform of Longitudinal Studies in Immigrant Families (PELFI) [24]. PELFI is a cohort study of 250 families: 82 from Ecuador, 82 from Colombia, 29 from Morocco, and 57 from Spain. In total, the cohort included 473 adults (190 men and 283 women). The baseline study was carried out in 2015, and two follow-up waves have been carried out to date (one in 2016 and another in 2017), with a family retention rate of 93.5% with respect to the baseline study.

### 2.2. Participant Selection

For this study, we used subjects selected from the PELFI cohort who met our inclusion criteria: families with an least one adolescent child aged 12 to 17, in which at least one adult age 18 to 65 resides, born in the same country (Spain, Colombia, Ecuador and Morocco) (in the case of biparental families, both parents must be from the same origin country), and family members must have a sufficient level of Spanish to respond to the interview questions. The definition of “family” was based on that used by the National Health Survey: people who occupy the same residence (or a part of it), who share a common budget (they share food costs or other costs that make up the budget), and who have lived together for at least six months at the time of recruitment [24].

For this analysis, adults were included who were working at the time of the interview or who had worked in the past seven days, even if for one hour, for money. In total the sample was *n* = 215 (31% Spaniards and 69% immigrants from Colombia and Ecuador).

### 2.3. Data Collection

We used questionnaires that collected information about adults. In order to collect sociodemographic variables, the baseline questionnaire was used. This questionnaire is made up of different modules: family composition and co-habitation, sociodemographic data, migratory process, social support, work situation, and state of health. To collect information on working conditions, the first follow-up questionnaire was used. This questionnaire has different parts: family composition and co-habitation, migratory process, work situation, domestic workload, reproductive care, general health, and consumption. And finally, the second follow-up questionnaire was used to collect information on dietary evaluation, which collects information related to family composition and co-habitation, general health, visual health, oral health, and nutritional health.

All of the parts of the questionnaires are made up of scales that come from other studies, with standardized instruments that measure the variables of interest such as diet [25] and employment conditions [26,27].

The full questionnaires can be found at http://www.ciberesp.es/programas-de-investigacion/subprogramas-estrategicos/subprograma-inmigracion-y-salud-ciberesp-sis-ciberesp.

### 2.4. Variables

#### 2.4.1. Explanatory Variables

Participants were divided into two groups by birth country: those born in Spain, and immigrants (born in Ecuador and Colombia). Immigrant acculturation was measured by time of residence, which is an indirect measure of acculturation used in other studies [28,29], and was categorized as ≥14 years and <14 years, where 14 years is the median number of years of residence. The principal explanatory variable was created based on the combination of these variables, migratory status, with three categories: born in Spain, immigrants (born in Colombia or Ecuador) with ≥14 years of residence, and immigrants with <14 years of residence.

The following employment conditions were also considered explanatory variables: work hours per week (≤40 h, >40 h), workday (regular: workday split into morning and afternoon, continuous morning workday and rotating shifts, except nights, irregular: continuous workday with afternoon/nighttime hours, continuous workday with night and early morning hours, rotating shifts, including nighttime shifts, irregular or variable workdays and others), and net monthly salary (≤751 EUR, >751 EUR).

The following socio-demographic variables were included: sex (woman/man), age (18–30, 31–40, ≥41 years), level of education (university studies, secondary studies, primary, or no studies), family type (biparental, single parent), and occupational social class (non-manual, manual), coded based on the 2011 Classification of Occupations [30].

#### 2.4.2. Outcome

Adherence to dietary recommendations was determined as a response variable, related to the consumption of certain food groups, and compliance with the nutritional guidelines of the Guide to the Mediterranean Diet used as the gold standard.

Dietary data were collected through the questionnaire of the frequency of food consumption (FFQ) used in the National Health Survey 2011/2012 [25]. This questionnaire consists of: 15 food groups (fruits, natural juice, meat, eggs, fish, pasta, bread, vegetables, legumes, sausages, dairy products, sweets, fast food, appetizers and sugary drinks) with their respective portion sizes, and nine categories of consumption frequency (+6 per day, 4–5 per day, 2–3 per day, 1 per day, 5–6 per week, 2–4 per week, 1 per week, 1–3 per month, never or <1 per month). Some modifications were introduced in both rations and frequency of consumption based on the FFQ used in other studies [31,32].

In order to analyze and interpret the results, the consumption frequencies were recoded into adequate and inadequate for each food group, according to the guidelines of the Guide to the Mediterranean Diet [33], with some modifications. Since the guide does not include recommendations for some food groups such as sugary drinks, fast food, and appetizers or salty snacks, the research team established recommendations based on consensus regarding other studies [34].

Adequate adherence was defined as; fresh fruit (excluding juices) (1 medium piece): ≥2–3 per day, natural fruit or vegetable juice (1 glass, 200 mL): ≤1 per day, meat (1 plate or portion), eggs (1), fish (1 plate or portion) and pasta, rice (1 medium plate), potatoes (1 medium potato): 2–4 per week, bread (1 small piece or 3 slices), grains (30 g): 4–5 per day, vegetables, salads (1 plate): ≥2–3 per day, legumes (1 medium plate): 2–4 per week, dairy products; milk (1 glass, 200 mL), cheese (1 portion, 50 g), yogurt (1, 125 g): 2–3 per day, sausages and cold cuts (1 portion, 50 g): ≤1 per week, sweets; cookies (1 cookie), pastries (1, 50 g), jam (1 teaspoon), cereal with sugar (30 g), candy (2, 30 g): ≤2–4 per week, sugary drinks (1): ≤1 per week, fast food; fried chicken (1 plate or portion), sandwiches (1), pizza (1 portion, 200 g), hamburger (1, 100 g) and appetizers or salty snacks (potato chips, cheetos, salted crackers) (1 bad, 50 g): ≤1–3 per month.

### 2.5. Data Analysis

A descriptive analysis was carried out of socio-demographic variables and working conditions by migratory status. Later, for each of the 15 food groups, we calculated the proportion of individuals with adequate adherence to dietary recommendations for Spaniards and immigrants based on time of residence in Spain. A logistic regression model was used for each food group to estimate the association between adequate adherence to dietary recommendations and the principal explanatory variable. Those born in Spain were considered the reference group. We controlled for the potential correlation in adherence to dietary recommendations within the same family by including a random effect by family in the logistic regression model [35]. The association measure used was the odds ratio, with the corresponding 95% confidence interval. To explore the influence of employment conditions on the association among those born in Spain and immigrants by time of residence with the different food groups, the following strategy was used: (1) A model was used that adjusted for socio-demographic variables (model 1), (2) Different models were used considering socio-demographic variables and each of the individual employment conditions variables (model 2: includes socio-demographic variables and work hours per week; model 3 includes socio-demographic variables and the type of workday; and model 4 includes socio-demographic variables and net monthly salary), and a final model that included all of the socio-demographic variables and employment conditions variables simultaneously (model 5). (3) The associations from model 1 were compared with the other models by calculating the percent change in the odds ratio. Comparing model 1 with the others allowed us to evaluate the influence for each of the employment conditions individually (model 2, model 3, and model 4) and simultaneously (model 5). In the cases in which the association would be inferior to the unit, odds ratios were obtained on a range from 1 to infinity, in order to do a better comparison between migratory status. The food groups “bread” and “grains” were excluded from the analysis because the frequency of adherence for the three groups was less than 1%. Stata version 10 (College Station, TX, USA) and SPSS version 15 (IBM, Armonk, NY, USA) were the statistical programs used to carry out the data management and statistical analyses.

### 2.6. Ethical Aspects

This study was carried out in accordance with national and international guidelines (Helsinki Declaration and Code of Ethics) and confidentiality regulations (Organic law 15/1999 on Protection of Personal Data). The project was approved by the ethics committee of the University of Alicante.

## 3. Results

Table 1 shows the distribution of the 215 participants (62.8% women). About 50.7% of the immigrants with less than 14 years of residence in Spain were under age 30, compared to 10.4% of Spaniards. For Spaniards, 41.8% had studied at university, compared to 16% of immigrants with more than 14 years of residence in Spain and 14.9% of immigrants with less than 14 years of residence in Spain. The percent of single parent families among immigrants was around 30%, and more than 90% were classified in manual occupations. Immigrants with less than 14 years of residence in Spain were the group most likely to work more than 40 h (22.4%) and to have salaries less than 751 euros per month. About 83.6% of Spanish workers reported working hours of less than 40 h, and 52.2% reported salaries greater than 750 euros per month.

Table 2 shows adherence to dietary recommendations for different food groups. Those born in Spain had greater adherence than did immigrants in terms of natural juices, eggs, fish, pasta, rice and potatoes, vegetables and legumes, dairy products, and sugary drinks. On the other hand, immigrants had greater adherence for the food groups sausages and cold cuts, and appetizers. For natural fruit or vegetable juice, Spanish adherence was nearly 100% (97.1%), with significant differences compared to immigrants with more than 14 years of residence (58%) and those with less than 14 years of residence (73.1%). This pattern was also observed in the case of pasta, rice, and potatoes (53.7% among Spaniards compared to 21% and 25.4% among immigrants). There are foods in which the adherence among Spaniards is different compared to immigrants, but the differences are less compared to those with more than 14 years of residence than to those with less than 14 years of residence. In the case of eggs (the adherence of Spaniards was 68.7% compared to 50.6% in immigrants with more than 14 years and 43.3% in immigrants with less than 14 years of residence), fish (adherence of Spaniards was 55.2% compared to 40.7% in immigrants with more than 14 years and 28.4% in immigrants with less than 14 years of residence), and vegetables and salads (adherence of Spaniards was 23.8% versus 12.4% in immigrants with more than 14 years and 9.0% in immigrants with less than 14 years of residence), dairy products (adherence among Spaniards was 38.8% compared to 13.6% in immigrants with more than 14 years and 9.0% in immigrants with less than 14 years of residence) and sugary drinks (adherence among Spaniards was 73.1% versus 45.0% in immigrants with more than 14 years and 37.3% in immigrants with less than 14 years of residence). In the case of appetizers or salty snacks, Spaniards (26.9%) and immigrants with more than 14 years of residence (35.0%) have similar levels of adherence, that are significantly less than those of immigrants with less than 14 years of residence (53.7%). These differences remain after controlling for the family effect using the logistic regression model.

The logistic regression models (Table 3) adjusted by socio-demographic and employment conditions show us that, in some cases, employment conditions can modify the association individually or collectively by 10%; however, there were no significant differences in the crude results. The multivariate analysis allowed us to explore in more detail the differences after adjusting by socio-demographic and employment conditions variables. Specifically, (1) there are foods for which there are no significant differences in adherence based on migratory status. These include fresh fruit excluding juices, meat, legumes, sweets, and fast foods. (2) There are foods for which there are statistically-significant differences in adherence of Spaniards compared to immigrants, with similar adherence among immigrants for both those residing in Spain for more than or less than 14 years. In these cases, Spaniards present greater adherence to dietary recommendations. These foods include natural fruit and vegetable juices (immigrants ≥ 14 years: 1/ORa = 25.60 and immigrants < 14 years: 1/ORa = 23.59) and pasta, rice and potatoes (immigrants ≥ 14 years: 1/ORa = 9.57 and immigrants < 14 years: 1/ORa = 9.62). (3) There are foods for which there are statistically-significant differences in adherence of Spaniards compared to immigrants with less than 14 years of residence, with smaller differences compared to immigrants with more than 14 years of residence, although these may be in some cases statistically significant. The cases in which Spaniards have greater adherence include eggs (immigrants ≥ 14 years: 1/ORa = 2.89 and immigrants < 14 years: 1/ORa = 3.92), fish (immigrants ≥ 14 years: 1/ORa = 2.33 and immigrants < 14 years: 1/ORa = 4.72), vegetables and salads (immigrants ≥ 14 years: 1/ORa = 3.26 and immigrants < 14 years: 1/ORa = 4.87), daily products (immigrants ≥ 14 years: 1/ORa = 14.34 and immigrants < 14 years: 1/ORa = 26.78), and sugary drinks (immigrants ≥ 14 years: 1/ORa = 2.12 and immigrants < 14 years: 1/ORa = 3.48). The case in which Spaniards have lower levels of adherence is that of sausages and cold cuts (immigrants ≥ 14 years: ORa = 7.62 and immigrants < 14 years: ORa = 24.65).

## 4. Discussion

These results show low adherence to dietary recommendations among the three groups. For most of the food groups, we observe a gradient in which adherence to dietary recommendations is greatest among Spaniards, followed by immigrants with more than 14 years of residence in Spain, and finally, by immigrants with less than 14 years of residence in Spain, except in the case of sausages and cold cuts, and appetizers and salty snacks, which shows the opposite tendency. After adjusting for socio-economic and employment conditions variables, we can see that these variables have limited influence on the results obtained.

The low adherence to dietary recommendations observed among Spaniards is consistent with the results of other studies carried out among the general population, which show that more than 60% of those surveyed reported a low adherence to the Mediterranean diet [34,36]. This could be due to changes in consumption patterns that are producing a “westernization” of the diet [37].

Acculturation is considered the greatest determinant of immigrant nutritional behavior, with different specific contexts by origin country and host country [38]. In our study, immigrants with more time residing in Spain show a general pattern of adherence to dietary recommendations that is similar to that of Spaniards, compared to immigrants with less time of residence. In the case of Spain and for the majority of food groups, acculturation seems to be a positive factor, given the greater adherence to the Mediterranean diet, which is considered an ideal diet in terms of quality and health [23,39], among immigrants with more time of residence. These results are along the lines of those of Marín-Guerrero et al., in which adherence to the Mediterranean diet increased among Latin American immigrants with their time of residence (>10 years), and became similar to those of the Spanish population [22]. Gallar et al. also observed a lower adherence to recommendations regarding fish in immigrants from Ecuador and Colombia, although this wasn’t the case for the rest of the food groups. These differences could be due to the fact that among the study population, 70% of the sample had resided in Spain for less than two years [19]. The low adherence observed in immigrants with shorter residence times could be explained by changes in food consumption patterns related to an increase in meat, high-fat foods, sweets, and refined carbohydrates in Latin American countries [28,40,41]. Our results do not agree with results obtained related to Latin American immigrants who live in Western countries, such as the United States. A systematic review showed that those immigrants who were least acculturated consumed more fruit, rice, beans, and less sugar and sugary drinks [14]. Another study, carried out among Latina women also showed that acculturation negatively affects nutritional habits among immigrants, with an increase in the consumption of salty snacks and fast food as the time of residence increases [42]. These differences among countries could be due to the influence of the diet of the host country.

In addition to adjusting by socio-demographic variables, our analyses also adjusted by working conditions variables, considered possible confounding factors in the relationship between migratory status and adherence to dietary recommendations. However, this adjustment did not affect the associations, given that the majority of the variables related to diet were shown not to be related to these variables. Other authors have also questioned the possible influence of these variables [12]. Other studies have evaluated employment conditions and diet related to nutrition habits rather than adherence to food groups, and they concluded that there is an association between shift work and long working hours and scant quantities of hot foods consumed during the day, snacking between mealtimes, and a lack of time for food preparation, among others [5,7,8].

This study includes a series of limitations. First, information about consumption of foods was self-reported. The disadvantage of this is a possible bias in self-reported data. Second, the measures of diet were carried out only once, which could result in errors in the estimates. However, this doesn’t imply a new FFQ measurement, which is a standardized instrument used in the national health surveys [25]. Furthermore, this was carried out by adding the standard sizes of portions and adjusting the consumption frequencies, using other FFQ validated for use in Spain among the adult population [31,32]. When comparing the intake of nutrients of these FFQ with that of the four dietary registries for one week, the average correlation coefficients for validity and reproducibility of the nutrient intake during one year were similar to other established dietary questionnaires [31,43]. Also, the literature reports the FFQ as an adequate measure for use in community studies, with good results in validating studies, and which permits obtaining global information about food consumption for comparison with dietary recommendations [44], which was the objective of this study. Even so, there is no need to suppose that such errors would differ in terms of migration status. In any case, the validity of instruments of measurement of diet may not be appropriate. Third, it is not known whether accurate results are possible using only a single indicator for acculturation, such as time of residence in the host country. Despite this, other authors have obtained similar results using other indicators such as attending school in the host country and speaking the language (MICASA) [12]. In our study, it is important to keep in mind that the immigrant participants were adults of Latin American origin, and therefore, they share a language with the host country. For this reason, we have not used other acculturation indicators. Quarter, the PELFI project [24], measured employment conditions at the time of the baseline survey and at the first follow-up. In this study, we’ve used working conditions as reported at the first follow-up, which were obtained one year prior to the data regarding food consumption, and which evaluated the usual consumption of food products over the last 12 months. However, comparing the changes in working conditions at the time of the baseline study to that of the first follow-up, 80% of individuals reported the same conditions. Also, the same analyses were carried out as in this study, using employment conditions at the time of the baseline study, and there were no significant differences in the results obtained using the conditions reported in the first follow-up, which reinforces the lack of effect of employment conditions in the pattern observed. Finally, the current study considered immigrants from Colombia and Ecuador, and therefore, the immigrant sample might not be representative of all immigrant workers in Spain in general, which would affect the external validity of the results obtained for other groups from other regions, with different socio-cultural characteristics. Nevertheless, Colombia and Ecuador are the most represented groups in Spain [18].

## 5. Conclusions

These results show that Spaniards do not have high adherence to dietary recommendations. However, Spaniards are followed by immigrants with more than 14 years of residence, and then by immigrants with less than 14 years of residence for the majority of food groups; natural fruit or vegetable juice, eggs, fish, pasta, rice and potatoes, vegetables and salads, dairy products and sugary drinks, except sausages and cold cults, appetizers and salty snacks, which shows the opposite tendency. Adjusting for socio-demographic variables and employment conditions variables has a limited influence on these results. Employment conditions can modify the association individually or collectively; however, there were no significant differences in the crude results. Therefore, employment conditions do not modify the effect of migratory status on adherence to dietary recommendations.

The challenge of this study lies in informing the design of strategies that aim to improve adherence to dietary recommendations for all groups of workers, but especially for immigrants residing in Spain for less time. It is also important to carry out additional qualitative studies that include immigrants from different countries of origin, in order to better understand the process of acculturation and its impact on diet.

## Figures and Tables

**Table 1:** Distribution of Workers Included in the Study by Socio-Demographic Characteristics and Employment Conditions by Migratory Status.

Variables	Born in Spain		Immigrant				Total	
	*n*	(%)	≥14 Years		<14 Years		*n*	(%)
			*n*	(%)	*n*	(%)		
socio-demographic								
Sex								
Woman	35	(52.2)	51	(63.0)	49	(73.1)	135	(62.8)
Man	32	(47.8)	30	(37.0)	18	(26.9)	80	(37.2)
Age (years)								
18–30	7	(10.4)	30	(37.0)	34	(50.7)	71	(33.0)
31–40	22	(32.8)	23	(28.4)	18	(26.9)	63	(29.3)
≥41	38	(56.7)	28	(34.6)	15	(22.4)	81	(37.7)
Level of education								
University studies	28	(41.8)	13	(16.0)	10	(14.9)	51	(23.7)
Secondary studies	30	(44.8)	49	(60.5)	47	(70.1)	126	(58.6)
Primary or no studies	9	(13.4)	19	(23.5)	10	(14.9)	38	(17.7)
Family type								
Biparental	58	(86.6)	56	(69.1)	43	(64.2)	157	(73.0)
Single parent	9	(13.4)	25	(30.9)	24	(35.8)	58	(27.0)
Occupational social class								
Non-manual	43	(64.2)	8	(9.9)	4	(6.0)	55	(25.6)
Manual	24	(35.8)	73	(90.1)	63	(94.0)	160	(74.4)
Employment conditions								
Work hours per week								
≤40	56	(83.6)	72	(88.9)	52	(77.6)	180	(83.7)
>40	11	(16.4)	9	(11.1)	15	(22.4)	35	(16.3)
Workday								
Regular	54	(80.6)	57	(70.4)	49	(73.1)	160	(74.4)
Irregular	13	(19.4)	24	(29.6)	18	(26.9)	55	(25.6)
Net monthly salary								
≤751 EUR	32	(47.8)	55	(67.9)	47	(70.1)	134	(62.3)
≥752 EUR	35	(52.2)	26	(32.1)	20	(29.9)	81	(37.7)
Total	67	(100.0)	81	(100.0)	67	(100.0)	215	(100.0)

**Table 2:** Frequency and Association (Odds Ratio with 95% Confidence Intervals) of Adherence to Dietary Recommendations for Food Groups by Migratory Status.

Variables	Born in Spain		Immigrant					Immigrant			
	*n*	(%)	≥14 Years		<14 Years		*p*	≥14 Years		<14 Years	
			*n*	(%)	*n*	(%)		ORc ^1^	(IC 95%)	ORc ^1^	(IC 95%)
Fresh fruit (excluding juices)	31	(46.3)	40	(50.0)	31	(46.3)	0.897	1.16	(0.61–2.22)	1.00	(0.51–1.97)
Natural fruit or vegetable juice	65	(97.1)	47	(58.0)	49	(73.1)	<0.001	0.02	(0.00–0.14) *	0.05	(0.01–0.29) *
Meat	24	(35.8)	26	(32.5)	24	(35.8)	0.888	0.89	(0.32–2.44)	0.97	(0.34–2.79)
Eggs	46	(68.7)	41	(50.6)	29	(43.3)	0.009	0.43	(0.18–1.02)	0.3	(0.12–0.76) *
Fish	37	(55.2)	33	(40.7)	19	(28.4)	0.007	0.55	(0.23–1.31)	0.29	(0.11–0.75) *
Pasta. rice and potatoes	36	(53.7)	17	(21.0)	17	(25.4)	<0.001	0.18	(0.07–0.46) *	0.23	(0.09–0.59) *
Bread and grains	0	(0.0)	5	(6.2)	0	(0.0)	0.012	-		-	
Vegetables and salads	16	(23.9)	10	(12.4)	6	(9.0)	0.049	0.45	(0.19–1.07)	0.31	(0.11–0.86) *
Legumes	29	(43.3)	48	(59.3)	29	(43.3)	0.077	2.04	(0.94–4.44)	1.01	(0.46–2.23)
Dairy products	26	(38.8)	11	(13.6)	6	(9.0)	<0.001	0.15	(0.05–0.48) *	0.09	(0.03–0.35) *
Sausages and cold cuts	20	(29.9)	45	(56.3)	45	(69.2)	<0.001	3.89	(1.52–9.92)	7.33	(2.54–21.12) *
Sweets	42	(62.7)	63	(77.8)	50	(74.6)	0.112	2.48	(0.97–6.30)	1.91	(0.74–4.88)
Sugary drinks	49	(73.1)	36	(45.0)	25	(37.3)	<0.001	0.27	(0.12–0.64) *	0.18	(0.70–0.49) *
Fast food	21	(31.3)	20	(24.7)	21	(31.3)	0.603	0.64	(0.22–1.85)	0.88	(0.30–2.64)
Appetizers or salty snacks	18	(26.9)	28	(35.0)	36	(53.7)	0.005	1.62	(0.62–4.26)	4.43	(1.57–12.51) *

^1^ Reference: Born in Spain; * *p* < 0.05.

**Table 3:** Association (Adjusted Odds Ratio (aOR) with 95% Confidence Intervals) of Adherence to Dietary Recommendations for Food Groups by Migratory Status, and change in association by employment conditions.

Variables	Immigrant									
	≥14 Years					<14 Years				
	aOR ^1^	(IC 95%)	1/aOR	Change aOR	Change 1/OR	aOR ^1^	(IC 95%)	a1/OR	Change aOR	Change 1/aOR
Fresh fruit (excluding juices)										
Socio-demographic	1.31	(0.58–2.94)				1.21	(0.50–2.92)			
Socio-demographics + work hours per week	1.30	(0.58–2.92)		−1%		1.22	(0.51–2.95)		1%	
Socio-demographics + workday	1.32	(0.58–2.99)		1%		1.21	(0.50–2.95)		0%	
Socio-demographics + net monthly salary	1.39	(0.61–3.18)		7%		1.29	(0.53–3.15)		6%	
Socio-demographics + employment conditions	1.38	(0.60–3.17)		6%		1.28	(0.52–3.16)		6%	
Natural fruit or vegetable juice										
Socio-demographics	0.05	(0.01–0.27) *	20.74			0.04	(0.01–0.26) *	23.61		
Socio-demographics + work hours per week	0.04	(0.01–0.25) *	22.99	0%	11%	0.04	(0.01–0.25) *	25.36	0%	7%
Socio-demographics + workday	0.05	(0.01–0.26) *	21.03	9%	1%	0.04	(0.01–0.26) *	23.49	8%	−1%
Socio-demographics + net monthly salary	0.05	(0.01–0.26) *	21.35	8%	3%	0.04	(0.01–0.26) *	24.34	4%	3%
Socio-demographics + employment conditions	0.04	(0.01–0.24) *	23.59	−3%	14%	0.04	(0.01–0.25) *	25.60	−1%	8%
Meat										
Socio-demographics	0.61	(0.17–2.14)	1.65			0.71	(0.19–2.73)	1.41		
Socio-demographics + work hours per week	0.60	(0.17–2.13)	1.66	−1%	1%	0.72	(0.19–2.78)	1.39	1%	−1%
Socio-demographics + workday	0.58	(0.17–2.06)	1.71	−4%	4%	0.70	(0.18–2.65)	1.44	−2%	2%
Socio-demographics + net monthly salary	0.59	(0.17–2.13)	1.68	−2%	2%	0.70	(0.18–2.71)	1.43	−2%	2%
Socio-demographics + employment conditions	0.56	(0.16–2.01)	1.79	−8%	9%	0.68	(0.18–2.65)	1.47	−4%	4%
Eggs										
Socio-demographics	0.35	(0.12–1.02)	2.82			0.26	(0.08–0.82) *	3.91		
Socio-demographics + work hours per week	0.33	(0.11–1.01)	3.02	−6%	7%	0.24	(0.07–0.83) *	4.11	−5%	5%
Socio-demographics + workday	0.36	(0.12–1.02)	2.81	0%	0%	0.26	(0.08–0.82) *	3.89	1%	−1%
Socio-demographics + net monthly salary	0.37	(0.13–1.06)	2.72	4%	−4%	0.27	(0.08–0.87) *	3.75	4%	−4%
Socio-demographics + employment conditions	0.35	(0.11–1.06)	2.89	−2%	2%	0.25	(0.07–0.88) *	3.92	0%	0%
Fish										
Socio-demographics	0.47	(0.16–1.41)	2.13			0.26	(0.08–0.90) *	3.81		
Socio-demographics + work hours per week	0.58	(0.15–1.44)	1.71	24%	−19%	0.25	(0.07–0.87) *	4.05	−6%	6%
Socio-demographics + workday	0.48	(0.16–1.40)	2.09	2%	−2%	0.26	(0.08–0.86) *	3.86	−1%	1%
Socio-demographics + net monthly salary	0.44	(0.14–1.36)	2.27	−6%	7%	0.24	(0.07–0.87) *	4.09	−7%	7%
Socio-demographics + employment conditions	0.43	(0.13–1.37)	2.33	−9%	10%	0.21	(0.06–0.78) *	4.72	−19%	24%
Pasta. rice and potatoes										
Socio-demographics	0.11	(0.03–0.35) *	9.09			0.11	(0.03–0.41) *	8.74		
Socio-demographics + work hours per week	0.11	(0.04–0.36) *	8.89	2%	0%	0.11	(0.03–0.41) *	8.85	−1%	0%
Socio-demographics + workday	0.11	(0.03–0.35) *	9.26	−2%	4%	0.11	(0.03–0.40) *	8.95	−2%	1%
Socio-demographics + net monthly salary	0.10	(0.03–0.33) *	9.67	−6%	9%	0.11	(0.03–0.39) *	9.36	−7%	6%
Socio-demographics + employment conditions	0.10	(0.03–0.34) *	9.57	−5%	8%	0.10	(0.03–0.38) *	9.62	−9%	9%
Vegetables and salads										
Socio-demographics	0.28	(0.10–0.83) *	3.54			0.19	(0.05–0.67) *	5.26		
Socio-demographics + work hours per week	0.29	(0.10–0.85) *	3.48	2%	0%	0.19	(0.05–0.66) *	5.34	−2%	0%
Socio-demographics + workday	0.28	(0.09–0.83) *	3.57	−1%	3%	0.19	(0.05–0.67) *	5.28	0%	−1%
Socio-demographics + net monthly salary	0.30	(0.10–0.92) *	3.29	8%	−6%	0.21	(0.06–0.74) *	4.84	8%	−9%
Socio-demographics + employment conditions	0.31	(0.10–0.94) *	3.26	9%	−6%	0.21	(0.06–0.75) *	4.87	8%	−9%
Legums										
Socio-demographics	2.61	(0.95–7.16)				1.30	(0.45–3.72)			
Socio-demographics + work hours per week	2.64	(0.95–7.33)		1%		1.30	(0.45–3.74)		0%	
Socio-demographics + workday	2.62	(0.95–7.19)		0%		1.30	(0.45–3.72)		0%	
Socio-demographics + net monthly salary	2.53	(0.91–7.03)		−3%		1.26	(0.44–3.66)		−3%	
Socio-demographics + employment conditions	2.56	(0.91–7.19)		−2%		1.25	(0.43–3.65)		−4%	
Dairy products										
Socio-demographics	0.08	(0.02–0.33) *	13.01			0.04	(0.01–0.22) *	24.37		
Socio-demographics + work hours per week	0.08	(0.02–0.34) *	12.79	0%	-2%	0.04	(0.01–0.22) *	24.25	0%	0%
Socio-demographics + workday	0.07	(0.02–0.32) *	13.38	−4%	3%	0.04	(0.01–0.21) *	24.87	−2%	2%
Socio-demographics + net monthly salary	0.07	(0.02–0.31) *	14.25	−10%	9%	0.04	(0.01–0.20) *	26.33	−8%	8%
Socio-demographics + employment conditions	0.07	(0.02–0.31) *	14.34	−11%	10%	0.04	(0.01–0.20) *	26.78	−9%	10%
Sausages and cold cuts										
Socio-demographics	8.66	(2.28–32.88) *				29.37	(6.00–143.66) *			
Socio-demographics + work hours per week	8.73	(2.30–33.13) *		1%		28.98	(5.95–141.29) *		−1%	
Socio-demographics + workday	8.06	(2.29–28.28) *		−7%		26.59	(6.06–116.65) *		−9%	
Socio-demographics + net monthly salary	7.65	(2.15–27.18) *		−12%		25.19	(5.67–111.96) *		−14%	
Socio-demographics + employment conditions	7.62	(2.15–27.07) *		−12%		24.65	(5.55–109.36) *		−16%	
Sweets										
Socio-demographics	2.32	(0.73–7.30)				1.59	(0.48–5.34)			
Socio-demographics + work hours per week	2.30	(0.73–7.27)		−1%		1.60	(0.48–5.37)		1%	
Socio-demographics + workday	2.31	(0.73–7.29)		0%		1.60	(0.48–5.40)		1%	
Socio-demographics + net monthly salary	2.21	(0.69–7.10)		−5%		1.52	(0.44–5.19)		−5%	
Socio-demographics + employment conditions	2.21	(0.69–7.12)		−4%		1.56	(0.45-5.34)		−2%	
Sugary drinks										
Socio-demographics	0.43	(0.15–1.28)	2.30			0.27	(0.08–0.91) *	3.65		
Socio-demographics + work hours per week	0.44	(0.15–1.32)	2.26	2%	−2%	0.27	(0.08–0.88) *	3.77	−3%	0%
Socio-demographics + workday	0.42	(0.14–1.26)	2.40	−4%	4%	0.27	(0.08–0.90) *	3.74	−2%	−1%
Socio-demographics + net monthly salary	0.47	(0.16–1.42)	2.11	9%	−8%	0.30	(0.09–0.99) *	3.36	8%	−11%
Socio-demographics + employment conditions	0.47	(0.15–1.47)	2.12	9%	−8%	0.29	(0.08–0.99) *	3.48	5%	−8%
Fast food										
Socio-demographics	1.05	(0.26–4.29) *				1.01	(0.23–4.51) *			
Socio-demographics + work hours per week	1.03	(0.25–4.25) *		−1%		1.02	(0.23–4.53) *		0%	
Socio-demographics + workday	1.05	(0.26–4.24) *		0%		0.99	(0.22–4.35) *		−3%	
Socio-demographics + net monthly salary	1.05	(0.25–4.32) *		0%		1.01	(0.23–4.53) *		0%	
Socio-demographics + employment conditions	1.03	(0.25–4.23) *		−2%		0.98	(0.22–4.39) *		−3%	
Appetizers or salty snacks										
Socio-demographics	4.63	(1.15–18.68) *				11.10	(2.44–50.42) *			
Socio-demographics + work hours per week	5.16	(1.12–23.79) *		11%		14.95	(2.84–78.75) *		35%	
Socio-demographics + workday	4.54	(1.13–18.17) *		−2%		10.66	(2.35–48.27) *		−4%	
Socio-demographics + net monthly salary	4.76	(1.16–19.51) *		3%		11.37	(2.47–52.26) *		2%	
Socio-demographics + employment conditions	5.31	(1.14–24.73) *		15%		15.08	(2.83–80.22) *		36%	

^1^ Reference: Born in Spain; Socio-demographic variables: sex, age, level of education, family type, occupational social class; * *p* < 0.05.

## References

[1] Lennernäs M., Hambraeus L., Akerstedt T. (1995). Shift related dietary intake in day and shift workers. Appetite.

[2] Bonham M.P., Bonnell E.K., Huggins C.E. (2016). Energy intake of shift workers compared to fixed day workers: A systematic review and meta-analysis. Chronobiol. Int..

[3] Zhao I., Turner C. (2008). The impact of shift work on people’s daily health habits and adverse health outcomes. Aust. J. Adv. Nurs..

[4] Morikawa Y., Miura K., Sasaki S., Yoshita K., Yoneyama S., Sakurai M., Ishizaki M., Kido T., Naruse Y., Suwazono Y. (2008). Evaluation of the effects of shift work on nutrient intake: A cross-sectional study. J. Occup. Health.

[5] Strzemecka J., Bojar I., Strzemecka E., Owoc A. (2014). Dietary habits among persons hired on shift work. Ann. Agric. Environ. Med..

[6] Bonnell E.K., Huggins C.E., Huggins C.T., McCaffrey T.A., Palermo C., Bonham M.P. (2017). Influences on Dietary Choices during Day versus Night Shift in Shift Workers: A Mixed Methods Study. Nutrients.

[7] Nicholls R., Perry L., Duffield C., Gallagher R., Pierce H. (2017). Barriers and facilitators to healthy eating for nurses in the workplace: An integrative review. J. Adv. Nurs..

[8] Sliwa S.A., Must A., Peréa F., Economos C.D. (2015). Maternal employment, acculturation, and time spent in food-related behaviors among Hispanic mothers in the United States. Evidence from the American Time Use Survey. Appetite.

[9] Norte A., Sospedra I., Ortíz-Moncada R. (2018). Influence of economic crisis on dietary quality and obesity rates. Int. J. Food Sci. Nutr..

[10] Linnan L., Arandia G., Bateman L.A., Vaughn A., Smith N., Ward D. (2017). The Health and Working Conditions of Women Employed in Child Care. Int. J. Environ. Res. Public Health.

[11] Porthé V., Ahonen E., Vázquez M.L., Pope C., Agudelo A.A., García A.M., Amable M., Benavides F.G., Benach J. (2010). ITSAL Project Extending a model of precarious employment: A qualitative study of immigrant workers in Spain. Am. J. Ind. Med..

[12] Matias S.L., Stoecklin-Marois M.T., Tancredi D.J., Schenker M.B. (2013). Adherence to dietary recommendations is associated with acculturation among Latino farm workers. J. Nutr..

[13] Satia-Abouta J. (2003). Dietary Acculturation: Definition, Process, Assessment, and Implications. Int. J. Hum. Ecol..

[14] Ayala G.X., Baquero B., Klinger C. (2008). A systematic review of the relationship between acculturation and diet among Latinos in the United States: Implications for future research. J. Am. Diet. Assoc..

[15] Norman S., Castro C., Albright C., King A. (2004). Comparing acculturation models in evaluating dietary habits among low-income Hispanic women. Ethn. Dis..

[16] Gilbert P.A., Khokhar S. (2008). Changing dietary habits of ethnic groups in Europe and implications for health. Nutr. Rev..

[17] Migration and Migrant Population Statistics–Eurostat Statistics Explained. http://ec.europa.eu/eurostat/statistics-explained/index.php?title=Migration_and_migrant_population_statistics/es.

[18] Instituto Nacional de Estadística. https://www.ine.es/jaxiT3/Datos.htm?t=9675.

[19] Gallar Pérez-Albaladejo M., Maestre J., Lillo Crespo M., Casabona Martínez I., Domínguez Santamaría J.M. (2007). Consumo de alimentos en inmigrantes de Elda y comarca. Cult. Los Cuid. Rev. Enferm. Humanidades.

[20] Neira-Mosquera J.A., Sanchez-Llaguno S., Pérez-Rodríguez F., Moreno-Rojas R. (2015). Assessment of the food patterns of immigrant ecuadorian population in southern spain based on a 24-h food recall survey. Nutr. Hosp..

[21] Gutiérrez Izquierdo M.I., Alarcón Rodríguez R., Latorre Fernández I. (2012). Hábitos alimentarios de la población inmigrante en Almería: Diferencias en el consumo de alimentos según procedencia. Metas Enferm..

[22] Marín-Guerrero A.C., Rodríguez-Artalejo F., Guallar-Castillón P., López-García E., Gutiérrez-Fisac J.L. (2015). Association of the duration of residence with obesity-related eating habits and dietary patterns among Latin-American immigrants in Spain. Br. J. Nutr..

[23] Willett W.C., Sacks F., Trichopoulou A., Drescher G., Ferro-Luzzi A., Helsing E., Trichopoulos D. (1995). Mediterranean diet pyramid: A cultural model for healthy eating. Am. J. Clin. Nutr..

[24] Cayuela-Mateo A., Martínez-Martínez J.M., Ferrer Serret L., Felt E., Casabona i Barbarà J., Collazos Sanchez F., Ronda-Pérez E., Cayuela-Mateo A., Martínez-Martínez J.M., Ferrer Serret L. (2017). Proyecto PELFI: Reclutamiento y características sociodemográficas de las familias inmigradas y autóctonas de las subcohortes de Alicante y Barcelona. Rev. Esp. Salud Pública.

[25] Ministerio de Sanidad, Consumo y Bienestar Social–Portal Estadístico del SNS–Encuesta Nacional. https://www.mscbs.gob.es/estadEstudios/estadisticas/encuestaNacional/encuesta2011.htm.

[26] Ronda E., Agudelo-Suárez A.A., García A.M., López-Jacob M.J., Ruiz-Frutos C., Benavides F.G. (2013). Differences in exposure to occupational health risks in Spanish and foreign-born workers in Spain (ITSAL Project). J. Immigr. Minor. Health.

[27] Ronda Pérez E., Benavides F.G., Levecque K., Love J.G., Felt E., Van Rossem R. (2012). Differences in working conditions and employment arrangements among migrant and non-migrant workers in Europe. Ethn. Health.

[28] Arcia E., Skinner M., Bailey D., Correa V. (2001). Models of acculturation and health behaviors among Latino immigrants to the US. Soc. Sci. Med..

[29] Esteban-Gonzalo L., Luis Veiga O., Gómez-Martínez S., Regidor E., Martínez D., Marcos A., Calle M.E. (2013). Adherence to dietary recommendations among Spanish and immigrant adolescents living in Spain; the AFINOS study. Nutr. Hosp..

[30] Domingo-Salvany A., Bacigalupe A., Carrasco J.M., Espelt A., Ferrando J., Borrell C. (2013). del Grupo de Determinantes Sociales de Sociedad Española de Epidemiología. Proposals for social class classification based on the Spanish National Classification of Occupations 2011 using neo-Weberian and neo-Marxist approaches. Gac. Sanit..

[31] Lopez J.V., Serra M.L., Aranceta B.J. (2006). Validez de la Evaluación de la Ingesta Dietética. Nutrición y Salud Pública: Métodos, Bases Científicas y Aplicaciones.

[32] Vioque J., Gonzalez L. Validity of a Food Frequency Questionnaire (Preliminary Results). https://journals.lww.com/eurjcancerprev/citation/1991/10001/validity_of_a_food_frequency_questionnaire.29.aspx.

[33] http://mediterradiet.org/dietamed/piramide_INGLES.pdf.

[34] Norte A., Ortiz-Moncada R. (2011). Calidad de la dieta española según el índice de alimentación saludable. Nutr. Hosp..

[35] Rabe-Hesketh S., Skrondal A. (2008). Multilevel and Longitudinal Modeling Using Stata, Volume II: Categorical Responses, Counts, and Survival.

[36] Cal Fernández M. (2017). Adherencia a la dieta Mediterránea en una muestra de la población adulta del sur de Galicia. Nutr. Clin. Diet. Hosp..

[37] Moreno L.A., Sarría A., Popkin B.M. (2002). The nutrition transition in Spain: A European Mediterranean country. Eur. J. Clin. Nutr..

[38] Raza Q., Nicolaou M., Snijder M.B., Stronks K., Seidell J.C. (2017). Dietary acculturation among the South-Asian Surinamese population in the Netherlands: The HELIUS study. Public Health Nutr..

[39] Zaragoza Martí A. (2015). Adherencia a La Dieta Mediterránea Y Su Relación Con El Estado nutricional en personas mayores. Nutr. Hosp..

[40] Barría R.M., Amigo H. (2006). Nutrition transition: A review of Latin American profile. Arch. Latinoam. Nutr..

[41] Albala C., Vio F., Kain J., Uauy R. (2001). Nutrition transition in Latin America: The case of Chile. Nutr. Rev..

[42] Teran-Garcia M., Morales-Perez M., Raffaelli M., Wiley A. (2011). Impact of acculturation on dietary habits of Latina immigrants. FASEB J..

[43] Willet W. (1998). Nutritional Epidemiology.

[44] Martin-Moreno J.M., Boyle P., Gorgojo L., Maisonneuve P., Fernandez-Rodriguez J.C., Salvini S., Willett W.C. (1993). Development and validation of a food frequency questionnaire in Spain. Int. J. Epidemiol..

